# Solar-powered oxygen, quality improvement and child pneumonia deaths: a large-scale effectiveness study

**DOI:** 10.1136/archdischild-2020-320107

**Published:** 2020-10-16

**Authors:** Trevor Duke, Francis Pulsan, Doreen Panauwe, Ilomo Hwaihwanje, Martin Sa'avu, Magdalynn Kaupa, Jonah Karubi, Eleanor Neal, Hamish Graham, Rasa Izadnegahdar, Susan Donath

**Affiliations:** 1 Intensive Care Unit, and Centre for International Child Health, Department of Paediatrics, University of Melbourne, The Royal Children's Hospital, Parkville, Victoria, Australia; 2 Discipline of Child Health, School of Medicine and Health Sciences, University of Papua New Guinea, Port Moresby, Papua New Guinea; 3 Department of Paediatrics, Wabag General Hospital, Wabag, Enga Province, Papua New Guinea; 4 Department of Paediatrics, Goroka General Hospital, Goroka, Eastern Highlands Province, Papua New Guinea; 5 Department of Paediatrics, Mendi General Hospital, Mendi, Southern Highlands, Papua New Guinea; 6 Department of Paediatrics, Mt Hagen General Hospital, Mt Hagen, Western Highlands Province, Papua New Guinea; 7 Infection and Immunity, Murdoch Childrens Research Institute, Parkville, Victoria, Australia; 8 Department of Paediatrics, University of Melbourne, Parkville, Victoria, Australia; 9 Bill and Melinda Gates Foundation, Seattle, Washington, USA; 10 Clinical Epidemiology and Biostatistics Unit, Murdoch Childrens Research Institute, Parkville, Victoria, Australia

**Keywords:** mortality, health services research

## Abstract

**Background:**

Pneumonia is the largest cause of child deaths in low-income countries. Lack of availability of oxygen in small rural hospitals results in avoidable deaths and unnecessary and unsafe referrals.

**Method:**

We evaluated a programme for improving reliable oxygen therapy using oxygen concentrators, pulse oximeters and sustainable solar power in 38 remote health facilities in nine provinces in Papua New Guinea. The programme included a quality improvement approach with training, identification of gaps, problem solving and corrective measures. Admissions and deaths from pneumonia and overall paediatric admissions, deaths and referrals were recorded using routine health information data for 2–4 years prior to the intervention and 2–4 years after. Using Poisson regression we calculated incidence rates (IRs) preintervention and postintervention, and incidence rate ratios (IRR).

**Results:**

There were 18 933 pneumonia admissions and 530 pneumonia deaths. Pneumonia admission numbers were significantly lower in the postintervention era than in the preintervention era. The IRs for pneumonia deaths preintervention and postintervention were 2.83 (1.98–4.06) and 1.17 (0.48–1.86) per 100 pneumonia admissions: the IRR for pneumonia deaths was 0.41 (0.24–0.71, p<0.005). There were 58 324 paediatric admissions and 2259 paediatric deaths. The IR for child deaths preintervention and postintervention were 3.22 (2.42–4.28) and 1.94 (1.23–2.65) per 100 paediatric admissions: IRR 0.60 (0.45–0.81, p<0.005). In the years postintervention period, an estimated 348 lives were saved, at a cost of US$6435 per life saved and over 1500 referrals were avoided.

**Conclusions:**

Solar-powered oxygen systems supported by continuous quality improvement can be achieved at large scale in rural and remote hospitals and health care facilities, and was associated with reduced child deaths and reduced referrals. Variability of effectiveness in different contexts calls for strengthening of quality improvement in rural health facilities.

**Trial registration number:**

ACTRN12616001469404.

**What is already known on this topic?**

- Pneumonia is the largest cause of death in children globally, and hypoxaemia is the major complication causing death.
- Oxygen therapy is life saving, but most health facilities in rural areas in low-income countries do not have reliable oxygen supplies.
- Many rural health facilities lack reliable power, and this limits the use of oxygen concentrators and other basic technology and provision of essential health services

**What this study adds?**

- It is feasible to scale up solar-powered oxygen therapy in rural health facilities in low-income and middle-income countries, and this requires multiple technical, clinical and management inputs and needs to be supported by continuous quality improvement.
- Solar-powered oxygen therapy and quality improvement in rural health facilities reduces death rates from pneumonia and other common conditions in children and reduces referrals.
- Variability of effectiveness of health interventions in rural health facilities and district hospitals calls for a stronger focus on quality improvement, education and problem solving, and much greater support for human resources.

## Introduction

Pneumonia is the leading cause of child deaths in low-income and middle-income countries, including in Papua New Guinea (PNG), a tropical country in the Western Pacific.^1 2^ Hypoxaemia is the major complication causing death in childhood pneumonia.^3 4^ In previous studies in provincial referral hospitals, we showed that improved oxygen systems, which include a reliable source of oxygen therapy using oxygen concentrators, and pulse oximetry for detection of hypoxaemia, can reduce mortality from pneumonia by up to 35%.^5^ However, in rural areas in low-income countries, most sick children present to district hospitals and health centres, where there are no reliable sources of therapeutic oxygen. Hypoxaemia occurs in many other common childhood conditions that present to primary health facilities: sepsis, febrile encephalopathy, bronchiolitis, asthma, tuberculosis, HIV-related lung disease, malaria, perinatal problems, trauma,^3 6–8^ as well as obstetric emergencies and acute and chronic respiratory and cardiac disease in adults.^9 10^ Therefore, reliable oxygen systems in rural health facilities may reduce deaths from pneumonia, reduce child mortality overall and greatly improve the way health clinics and hospitals serve the needs of communities.

A major barrier to improving oxygen systems in rural and remote settings has been unreliability of power, such that concentrators are unable to function because of limited power and are damaged by surges in power. Other barriers include the long-term reliability of medical devices in such settings, costly and challenging logistics of installation of health infrastructure in regions where roads are poor or in remote islands. Lack of preventative maintenance and capacity for repairs is also a barrier to health technology in remote settings. Furthermore, there has been a lack of recognition of the importance of oxygen as an essential service, difficulties identifying hypoxemia without pulse oximetry, limited training of health workers in the recognition and management of hypoxaemia and lack of financial investment in quality health services.

In PNG as in many countries, there is a significant child mortality gradient between rural and urban areas. To address this, health infrastructure and quality need to be improved, and oxygen and power are components of this. We report the results over 7 years of a large-scale study of solar-powered oxygen systems in 38 rural health facilities in nine provinces of PNG with a total population of over 1.3 million, where we aimed to overcome these barriers.

## Method

The implementation of this programme has been described in detail elsewhere.^11^ In brief, we implemented a programme for improving reliable oxygen and sustainable power in 38 remote health facilities, equipment used included Airsep Elite 5 L/min concentrators (Chart Industries, New York), Lifebox pulse oximeters (www.lifebox.org), oxygen analysers (Maxtec O_2_ analyzer, Utah, USA) to monitor the performance of concentrators, 4.9 kW solar power system using 18 240 W solar panels, which was designed to produce on average 11.44 kWh/day power and a battery back-up, designed to provide 3 days’ autonomy at 80% depth of discharge (see online supplemental appendix I).^11 12^ Initial steps included community engagement, selection of participating health facilities, the specifications and design of the solar and oxygen systems, healthcare worker training, engineering and maintenance capacity, logistics and programme implementation.^11^ Community engagement involved meetings with provincial and district health staff to understand needs at the health facility level and where oxygen fitted into priorities, and to hear the experiences of staff in caring for children with pneumonia and the potential effect of improving oxygen on referral patterns. We selected health facilities on the basis of high community burden of pneumonia, lack of reliable source of oxygen, limited or unreliable power, and staff being committed and enthusiastic to participate. The roll-out of the intervention occurred in 2015–2017: most of the facilities were covered throughout 2015 to March 2016, two in late 2016 and three in 2017.

10.1136/archdischild-2020-320107.supp1 Supplementary data

### The settings

The 38 health facilities had wards for overnight care of children. Most were in rural districts. The catchment population of the health facilities was 1.3 million, out of a total PNG population of 8.8 million. The median number of beds was 10 for children and 4 for newborns. Twelve of the health facilities had doctors. All health facilities had one or more nurses: the median number was four. Eighteen of the health facilities had a trained paediatric nurse, and the rest were general trained nurses. All health facilities had one or more community health workers (median number of seven).

### Training and continuous quality improvement

We used the WHO guidelines for the Clinical Use of Oxygen in Children^13^ and the WHO Hospital Care for Children training.^14^ The training was practical, repeated on several occasions (typically annually in a regional training course, but this involved different healthcare workers from these facilities each time) and was reinforced on health centre visits by the provincial paediatricians. The content and delivery of the training is described in a previous paper but covered pneumonia care, oxygen therapy, the clinical management of other common illnesses in newborns and children, monitoring and supportive care.^11^ We taught healthcare workers how to monitor the performance of oxygen concentrators using oxygen analysers and about regular weekly maintenance. Training was hands-on and practical and emphasised continuous improvement, sharing of experiences and problem solving. Further details of the continuing education programme is described in online supplemental appendix II. Quality improvement was supported by district visits from the paediatrician and a technician trained in oxygen and solar equipment every 4–6 months and involved on-site training, troubleshooting of identified problems, facilitation of local problem solving, audit and feedback.

10.1136/archdischild-2020-320107.supp2 Supplementary data

### Data collection and sample size

We gathered data from each of the health facilities on all admissions for children aged birth to 13 years of age: number of admissions and number of deaths, number of pneumonia admissions and deaths, number of neonatal admissions and number of deaths, and number of referrals. Data were collected per year for each facility. The intervention was introduced in 2015–2017; most of the facilities were covered throughout 2015 to March 2016, two in late 2016 and three in 2017. Preintervention data were for the years 2012–2016 depending on when the intervention was introduced in the health facility. Postintervention data were gathered from the time of intervention installation in each facility until July–August 2019, when the last round of health facility visits was conducted.

The data were collected from the health facility admission and discharge record books, which are generally kept meticulously by senior nursing staff in PNG. Each facility has a record book, and details of every admission is entered manually; the data include patient name, contact address, diagnosis at admission and discharge, and outcome.

The primary outcome was a difference in mortality from pneumonia between preintervention and intervention eras. Secondary outcomes were overall paediatric mortality, neonatal mortality and rate of referrals.

In the process of initial engagement with health facilities and gathering baseline data, we assessed the quality of data. Initially, we gathered data from seven additional health facilities, which did not receive the infrastructure intervention, often for several reasons: poor road conditions that would not allow a vehicle carrying equipment to access the health centre (n=3); the health centre was in a state of disrepair (n=3); and there was inadequate baseline data quality (n=7).^11^ These seven health facilities were excluded and other health facilities that fulfilled the criteria for participation were chosen to replace them; hence, some facilities received the intervention later, in 2016 and 2017. Data availability and quality were assessed throughout; the data on a given health facility for a given outcome were used in the analysis if the admission record book was complete and kept up to date. To do this, we checked to see there were no missing pages or dates, such as weeks or months missing when there were no entries and no other explanation (such as the health facility was closed for a period), and that basic data: name, date of birth/age, diagnoses and outcomes were all filled in. Because some facilities had gaps in data quality over the period of the study, only health facilities that maintained good quality data in the preintervention and postintervention eras were included in each analysis (pneumonia deaths, overall paediatric mortality, neonatal deaths and referrals). Thus, the number of health facilities with data that could be used for each analysis differed (table 1 and Results).

**Table 1:** Summary of admissions and deaths for all children, pneumonia, births, neonatal deaths and referrals

	Total paediatric admissions 37 hospitals	Paediatric deaths 37 hospitals	Pneumonia admissions 36 hospitals	Pneumonia deaths 36 hospitals	Births 35 hospitals	Neonatal deaths 35 hospitals	No of paediatric referrals 35 hospitals
Pre-intervention	31 158	1392	10 228	377	33 633	370	2262/21 455 *
Post-intervention	27 166	867	8705	153	30 776	349	1062/22 235 *
Total	58 324	2259	18 933	530	64 409	719	3397

*Total admissions denominator is different because the two provincial referral hospitals were excluded from this analysis.

### Cost analysis

Detailed costs of the intervention were kept throughout the project (table 2). These included costs of equipment and infrastructure, logistics and installation, clinical, technical and quality improvement training and maintenance. While there were economies of scale, we averaged costs for an individual health facility to provide information on the indicative cost to conduct this intervention out in any given health facility. The cost per life saved was calculated as the overall costs divided by the estimated number of deaths avoided.

**Table 2:** Programme costs overall and for one health facility

Item	Item cost (US$)	Number	Total cost (US$)
Solar and oxygen equipment			
Solar power system, including battery	41 000	1	41 000
Nasal oxygen prongs	3	200	600
Oxygen concentrators – 5 L/min	648	3	1944
Sureflow flowmeter Airsep	550	1	550
Lifebox pulse oximeter	250	3	750
Oximeter sensor probes (five per oximeter)	30	15	450
Uninteruptable power supply units	200	2	400
Oxygen analyser: flow and concentration	385	1	385
Paediatric cannula, 7 feet	3	100	300
Cannula tubing, 25 feet	6	100	600
Intermediate infant cannula 7 feet	3	100	300
Neonate cannula, 7 feet	3	100	300
Cannula tubing, 15 feet	4	100	400
Connectors	1.50	5	8
Spare parts for oxygen concentrators			
Filter, internal	12.50	1	13
Filter, external foam	3	1	3
Sieve beds 5 L	185	1	185
Compressor 220 v	180	1	180
Circuit board concentrator	170	1	170
Logistics and installation			
Delivery of equipment to health centre	3000	1	3000
Installation costs	2000	1	2000
Cost per health centre			53 537
Overall programme costs			
Sea freight	18 000		
Air freight	27 000		
Vehicle fuel and maintenance	10 000		
Training – clinical, technical and quality improvement	150 000		
Total programme costs	205 000	/38 *	5395
Total cost per health facility			58 932
Overall costs			2 239 416

* total number of participating health facilities

### Other data collected

We also gathered data on healthcare workers perceptions of the programme, and maintenance requirements of the equipment, and these will be reported elsewhere.

### Statistical analysis

For each health facility, data were available for up to 4 years preintervention and postintervention (details in online supplemental appendix II). For each variable collected, the average number per year preintervention and postintervention was calculated by hospital; these averages were used in all analyses. For the primary and secondary analyses, we included health facilities if they had good quality data from both preintervention and intervention eras for that outcome.

For each outcome, Poisson regression with random intervention effects^15^ was used to estimate the overall incidence rate ratio (IRR): the incidence rate (IR) postintervention divided by the IR preintervention, and the overall IR preintervention and postintervention. This method allows for inclusion of data from all health facilities, including those where the preintervention or postintervention IR was zero and also allows for heterogeneity in the effect of the intervention between health facilities.

We estimated the number of lives saved in the postintervention period by the difference between observed and expected mortality. Expected mortality was based on mortality in the preintervention period, and the number of deaths averted in subsequent years of observation was calculated by applying the same mortality IR as in the preintervention period and the IRR. Thus: number of deaths avoided = (preintervention IR × postintervention admissions) – (preintervention admissions × IRR × preintervention IR).

Data analysis was conducted using Excel (Microsoft) and Stata version 16.1.

### Sample size calculations

The sample size calculation was based on an anticipated 25% reduction in the primary outcome of pneumonia mortality, from an estimated baseline of 4%–3%, with 90% power, requiring 7295 children with pneumonia in each arm. The study was also adequately powered to detect a 20% reduction in both overall paediatric mortality and referrals to tertiary centres.

### Ethics approval and consent to participate

The project was registered by Australian New Zealand Clinical Trials Registry. The study protocol is published.^11^ The data collection was based on routine reporting, so no individual patient consent was required by the ethical review committees.

## Results

Table 1 shows the admissions, deaths for the preintervention and postintervention eras, overall, for pneumonia, births and neonatal deaths, and referrals. On-line appendix III shows the data for each health facility.

10.1136/archdischild-2020-320107.supp3 Supplementary data

### Pneumonia admissions and deaths

Thirty-six health facilities had good quality preintervention and intervention data for pneumonia admissions and deaths. In the preintervention era, there were 10 228 pneumonia admissions and 377 pneumonia deaths (IR 2.83 deaths per 100 pneumonia admissions, 95% CI 1.98 to 4.06). In the postintervention era, there were 8705 pneumonia admissions and 153 pneumonia deaths (IR 1.17 deaths per 100 pneumonia admissions, 95% CI 0.48 to 1.86). Pneumonia case numbers were significantly lower in the postintervention era than in the preintervention era: a median of 63 pneumonia cases (IQR 24–134) in the preintervention era and 37 (IQR 14–87) in the postintervention era. The estimated IRR for pneumonia deaths postintervention was 0.41 (0.24–0.71, p<0.005). If the pre-era mortality and IRR are applied to the postintervention era, there were 145 pneumonia deaths averted in the first 3 years [(0.0283×8705) – (0.41×0.028 × 8705)].

**Figure 1:**
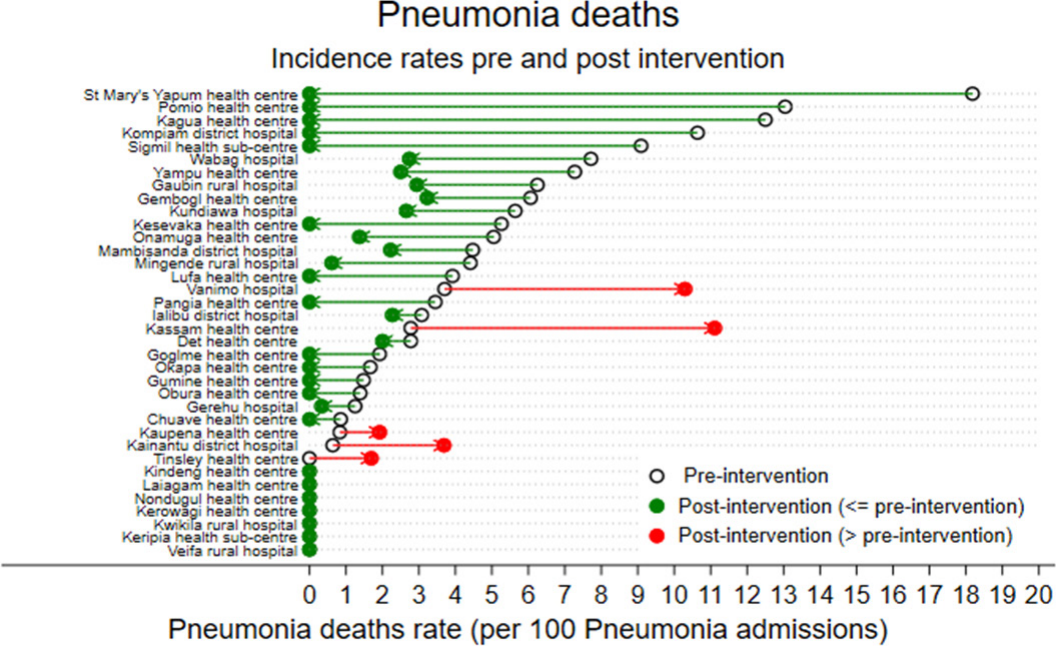
Change in incidence rate of pneumonia deaths in all health facilities.

Figure 1 shows the change in incidence of pneumonia deaths in all health facilities. There was heterogeneity in outcomes between facilities, including five health facilities where the pneumonia death IR increased.

### Overall paediatric admissions and deaths

**Figure 2:**
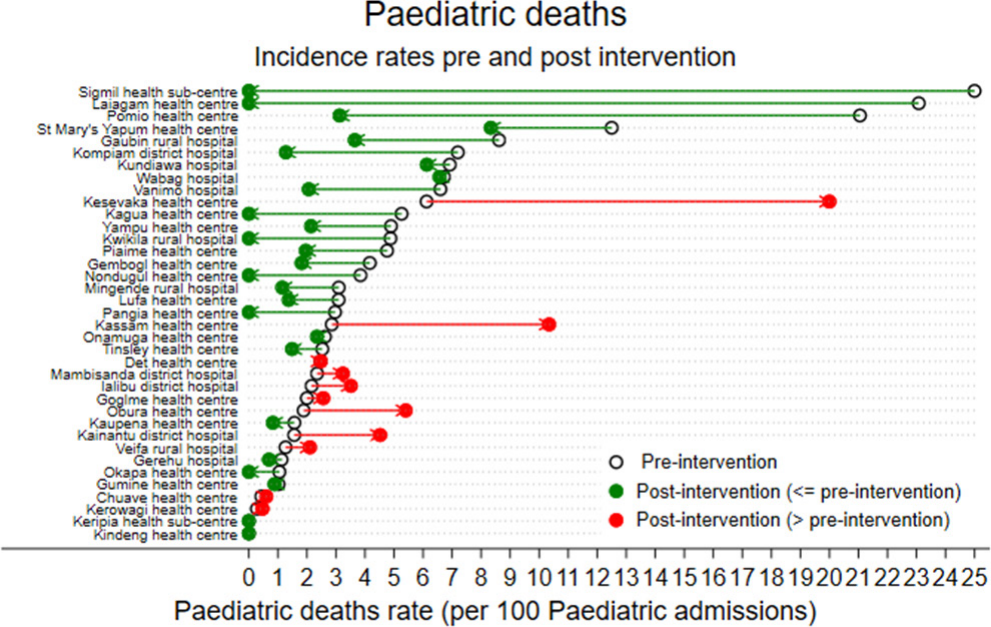
Change in incidence rate of paediatric deaths in all health facilities.

Thirty-seven health facilities had good quality preintervention and intervention data for overall paediatric admissions and deaths. In the preintervention era, there were 31 158 paediatric admissions and 1392 deaths (IR 3.22 deaths per 100 paediatric admissions, 95% CI 2.42 to 4.28). In the postintervention era, there were 27 166 admissions and 867 deaths (IR 1.94 deaths per 100 paediatric admissions, 95% CI 1.23 to 2.65). Paediatric case numbers were similar in the preintervention and postintervention eras: a median of 115 (IQR 72–309) in the preintervention era and median of 121 (IQR 34–263) in the postintervention era. The estimated IRR for paediatric deaths postintervention was 0.60 (0.45–0.81, p<0.005). If the pre-era mortality rate was applied to the postintervention era, there were 348 deaths averted in the first 3 years [(0.0322×27 166) – (0.6×0.0322 × 27 166)]. Figure 2 shows the change in incidence of paediatric deaths in all health facilities. Again there was variability in effectiveness by site.

### Referrals

Thirty-five health facilities had good quality preintervention and intervention data for referrals; two were excluded as they were provincial hospitals that were the recipient hospital of referral from health centres and district level hospitals, not from where onward referrals were made. These two provincial hospitals made no onward referrals in the intervention era. Another health centre was excluded because of inadequate data quality on referrals. In the preintervention era, there were 2262 children referred from these 35 health facilities to provincial or tertiary hospitals (10.5% of all 21 455 paediatric admissions from these 35 health facilities). The preintervention IR for referrals was 11.29%. In the postintervention era, there were 1062 referrals (4.8% of all 22 235 paediatric admissions in these 35 health facilities). The postintervention IR for referrals was 4.14 (2.29–6.00). The estimated IRR postintervention was 0.37 (0.25–0.53). Based on the preadmission rate of referrals, the number of referrals avoided was 1582 (0.113 × 22 235 – (0.37×0.113 × 22 235).

### Births and neonatal deaths

For births and neonatal deaths, 35 health facilities had good quality data in the preintervention and intervention eras. In the preintervention era, there were 33 633 births and 370 neonatal deaths (mortality rate 12.3 neonatal deaths per 1000 births, 95% CI 11.2% to 13.5%). In the postintervention era, there were 30 776 births and 349 neonatal deaths (mortality rate 11.5 deaths per 1000 births (95% CI 10.4 to 12.8). The estimated preintervention and postintervention IR were 7.9 per 1000 births (5.7–10.9) and 8.8 per 1000 births (5.5–12.1), respectively. The estimated IRR for neonatal deaths postintervention was 1.11 (0.76–1.62, p=0.58).

### Costs

It is relatively inexpensive to provide oxygen concentrators and pulse oximeters; however, solar power is expensive, especially in remote areas, as are implementation costs. The cost per health facility was approximately US$58 932, including oxygen and solar equipment, installation and logistics, maintenance and training over 4 years (table 2). Over the course of the formal training, on-site support and continuous quality improvement, all health workers who manage children in these 38 facilities had training and hands-on assessment of skills.

The total cost of US$2 239 416 (average of $58 932 in each of the 38 health facilities) means that cost per paediatric death averted was $6435.

## Discussion

How to reduce the high rates of pneumonia mortality among children in remote and rural settings in low-income countries, how to achieve sustainable energy and green health facilities and how to improve quality of healthcare for poor communities are three big issues in health globally. This study showed that a solar-powered oxygen programme supported by quality improvement was associated with reductions in overall paediatric mortality by 40%, mortality from pneumonia by over 50% and a reduction in referrals from health centres and district hospitals across nine rural provinces in PNG. The broader message is that investment in basic infrastructure, training and quality improvement can save lives in low-income and middle-income countries, even in remote areas.

The infrastructure included oxygen concentrators, pulse oximetry, oxygen analyers and solar power. While previous studies have shown oxygen in referral hospitals in developing countries improve outcomes,^5 16 17^ no study has been done at large scale involving primary healthcare in district healthcare settings, where mortality has been measured. Quality improvement inputs included training in clinical paediatric care and oxygen therapy, small group problem solving and sharing of experiences between healthcare workers, audit and feedback. The quality improvement approach as well as the appropriate infrastructure enabled most health facilities to improve; in the absence of quality improvement in health systems, technology is likely to fail or be suboptimal in effectiveness.

There was variability in the effectiveness of the intervention by health facility, while most health facilities demonstrated a reduction in mortality from pneumonia and overall paediatric deaths, 5 health facilities experienced an increase in the IR for pneumonia and 11 health facilities experienced an increase in the overall paediatric mortality. The numbers of cases contributed by each of these facilities individually were small and therefore prone to IR changes with one or two additional deaths. This is except in one larger district hospital (Kainantu) where case ascertainment of deaths may have been greater in the postintervention era, and the postintervention pneumonia mortality was 3.8% out of nearly 1000 pneumonia admissions, comparable with many good medium-sized provincial hospitals. It is a fact however that the intervention was not as effective in some health facilities as it was others, and we identified some health facilities early as not functioning well enough to sustain this intervention. The reasons are varied, which underlines that a quality improvement approach is needed, where local gaps can be identified and addressed, and emphasises that there are basic health system and health facility pre-requisites for any health care initiative to succeed.

Our study was a before-and-after study. The limitations of the design include the possibility of secular trends in case numbers and mortality rates over time, ascertainment bias and altered thresholds for hospital admission. We took many steps to avoid bias, including using the same mechanism of data collection in the pre-era and post-era and using the most complete data collection method possible. Clinical staff knew about the evaluation of the solar oxygen system; however, they did not know that outcome data were derived from ward admission record books. The accuracy with which data were recorded did not change during the study. There were significantly fewer children with pneumonia admitted in the postintervention era, and this requires some consideration.

Among interventions that may have reduced child pneumonia numbers and overall child deaths in PNG, the *Haemophilus influenza* type b conjugate vaccine was introduced in 2008, and the pneumococcal conjugate vaccine (PCV-13 valent) was introduced in 2014. Coverage was very low in the early years for PCV-13; an estimated 20% of children had received PCV-13 in 2015 and 2016. This increased to 36% in 2017 and remained static at 35% in 2018–2019.^18^ PCV coverage rates may even be overestimated as they are based on DTP-3 data, the assumption being that all children receiving DTP-3 will also have received PCV, as it is given at the same time. At least in the early years, distribution of PCV-13 was not as uniform as DTP. Independent evidence from the PNG Demographic and Health Survey 2016–2018 indicated only 35% of children had received all basic vaccinations by 12–23 months of age (including PCV-13).^19^ We do not have specific data on PCV-13 coverage from the districts in which this study was conducted, and in these remote districts lower than national average, coverage is likely. However, conjugate vaccines may have changed the proportion of children with bacterial pneumonia and lowered the risk of mortality in the era 2016–19 compared with 2012–15.

Other secular trends, such as overall falls in child mortality, may affect preintervention and postintervention studies conducted over long periods of time. Comparison of the Demographic and Health Surveys data between 2006 and 2016–2018 shows a reduction in estimated overall child mortality (74 to 49 per 1000 live births) and infant mortality (57 to 33 per 1000 live births) in PNG.^19^ It is possible that this oxygen programme contributed to that reduction given the scale of the project, involving health facilities serving 1.3 million people, about 14% of the 8.8 million population of PNG, and the regions where child mortality has historically been highest. However these overall improvements in child mortality may partly account for some of the outcomes we observed.

We did not collect other demographic data that may help explain the trends observed; however, PNG DHS provide data on health-related behaviour, social and economic changes. Between 2006 and 2016–2018, there was no evidence of an improvement in care seeking for an acute respiratory infection in the country overall, and the proportions of women delivering in a health facility, and breastfeeding rates, were both unchanged.^19^ Parental educational attainment improved in PNG in that time, and mobile telephone ownership has increased markedly. While these may partly account for the lower overall child mortality rates in that time, there were few other changes over the years 2006 to 2016–2018 that might influence pneumonia rates or care seeking. Housing materials remained similar; water and sanitation in rural areas remained unimproved for the majority of rural populations.^19^ Rural communities, including those in this study, remain far more disadvantaged in every statistic. Advances in health outcomes are aggregates of progress in many sectors; new vaccines, contemporaneous economic and social advances may be responsible for much of the effects observed in this study, but health service improvement from this intervention also likely played a key role.

Previously oxygen has been thought of as an essential drug,^20^ but it is also an essential *service*. With the drive to increase facility births in low-income, high-mortality countries, health facilities have to reach a certain standard to signal to the community that they can provide a greater level of service than delivering at home; these essential services include power, clean water, oxygen, infection control and prevention, and sanitation.

Sustainability and expansion of such programmes require an accurate estimation of true costs and benefits, so that health administrators and governments can order priorities according to their needs and budget according to available resources. In a decentralised system where local health authorities have to decide on their health spending priorities, this project identified the full costs of such a health facility service upgrade. The estimated cost per death averted (US$6435) is more than the cost estimated in 2008 (US$1673 per life saved) in provincial-level hospitals, where power was generally reliable.^5^ Renewable power and logistics in remote areas add to the cost. However, renewable power also provided an unanticipated solution to the common problem of oxygen concentrators being damaged by frequent power surges (power surges do not occur with solar power). Moreover, solar power provided for other aspects of health system quality such as light and security. In many of the health facilities, a solar-powered light enabled for the first time greater safety of births at night. Other simple things, such as a health worker being able to recharge her mobile phone, and therefore communicate with a provincial paediatrician regarding a sick child, were enabled by the solar power. It is likely that some of the effect of the project on overall paediatric mortality was not from oxygen per se but the benefits of reliable power to the health facilities. Over 1500 referrals were avoided; the cost saved from this is hard to estimate, as cost saved from a referral differs markedly by family economic circumstances, geography, mode of transport and length of referral hospital stay. However, the transport costs are high from remote areas, deterioration in transit for a critically ill child is a common occurrence and the costs to families of being hospitalised away from their community are high. So the broader cost savings, for the health service, communities and families, are very substantial.

There are very few recent analyses of cost per life saved from other pneumonia interventions. Early estimates of pneumococcal vaccine cost per life saved was US$4500 for countries with under-5 mortality rates 25–99 per 1000 live births,^21^ US$847–US$2555 in several South American countries^22^ and over US$22 000 in Brazil.^23^ Only estimates of community case management of pneumonia are lower, with published modelling estimates of US$150–350 per life saved.^24^ For community case management to be optimally effective, there must be district health facilities with effective basic infrastructure and quality care to deal with sicker children who need oxygen, monitoring and a level of care beyond mere provision of antibiotics.

Sustainability of such projects depends on a structure, capacity and resourcing for quality improvement. This includes technical capacity (such as skills in renewal and maintenance of infrastructure and equipment), clinical capacity including continuing professional development for healthcare workers, financing of rural health services to meet basic needs and development of simple data systems that can be used for quality improvement initiatives. There are many locations in the world where such health system improvements are needed.

## Conclusions

In low-income countries, solar-powered oxygen and a continuous quality improvement programme may reduce child mortality and deaths from pneumonia, allow healthcare to be provided closer to the community in which people live and be transformational for rural health services. However, this will only be effective if quality improvement can identify and address gaps and support staff to improve. Clinical quality improvement requires increases in human resources, education and structure, and these need strengthening in most low-income and middle-income countries.
