# Supplementary material for: Solar-powered oxygen, quality improvement and child pneumonia deaths: a large-scale effectiveness study
Source: Arch Dis Child. 2020 Oct 16;106(3):224–30. doi: 10.1136/archdischild-2020-320107 (PMC7907560; doi:10.1136/archdischild-2020-320107)
Supplement: Supplementary data [file archdischild-2020-320107supp002.pdf]

Online appendix II

Training course in Hospital Care for Children and Quality Improvement

Day 1 - Monday - Respiratory disease, oxygen therapy, the stages of management of any sick child

Time	**Activity**	**Presenter**
0830-0845	Opening, Welcome	
0845 – 0915	Introduction to the Hospital Care for Children program<br>Introductions of participants and facilitators<br>Aims and objectives of the workshop:<br>• To be able to use the Hospital Care for Children guidelines in everyday clinical practice<br>• To understand the stages of management of any sick child<br>• Train in the use oxygen concentrators and pulse oximeters<br>• Train in quality improvement in pediatric care	
0915-1030	Cough and difficult breathing, clinical case II<br>Child with severe respiratory distress<br>The stages of management of any sick child	
1030 – 1100	Tea break	
1100-1130	Respiratory case videos	
1130 -1300	Practical, hand-on training in oxygen concentrators, oximeters, oxygen analysers.	Biomedical engineer
1300-1400	Lunch break	
1400-1600	Practice on the paediatric ward<br>Small groups, coaching method of teaching<br>Problem solving	All facilitators, small groups of 4-5
1600 – 1630	Tea break	
1630-1630	Recap, discussion and questions	

Duke T, *et al. Arch Dis Child* 2021; 106:224–230. doi: 10.1136/archdischild-2020-320107

Online appendix II

Day 2 – Tuesday – Diarrhoea, Fever and Malnutrition

**Time**	**Activity**	**Presenter**
0830-0930	Diarrhoea and dehydration	
0930-1030	Fever, clinical case	
1030 – 1100	Tea break	
1100 – 1230	Severe malnutrition, clinical case	
1230- 1330	Lunch break	
1330-1600	Clinical practice using the WHO guidelines in Paediatric ward<br>Small groups, coaching method of teaching, problem solving<br>Practical oxygen concentrator teaching in ward	All facilitators, small groups of 4-5
1600-1630	Recap, discussion and questions	

Online appendix II

Day 3 – Wednesday – Neonatal Care

**Time**	**Activity**	**Presenter**
0815-0830	Neonatal illness in Papua New Guinea - overview	
0830-0900	Early Essential Newborn Care	
0900 – 0945	Young infants with infections, clinical case	
0945 – 1030	The low birth weight baby, clinical case	
1030 - 1045	Tea break	
1045 – 1200	Birth asphyxia, clinical case<br>Practical training: Neonatal resuscitation and Early Essential Newborn Care<br>Coaching method of teaching	
1200-1230	Clinical signs of serious neonatal illnesses	
1300 - 1400	Lunch break	
1400 - 1600	Clinical practice in Neonatal Ward<br>Safe oxygen use in neonatal care<br>Problem solving	All facilitators, small groups of 4-5
1600 - 1630	Recap, discussion, sharing of experiences	

Duke T, *et al. Arch Dis Child* 2021; 106:224–230. doi: 10.1136/archdischild-2020-320107

Online appendix II

Day 4 – Thursday - HIV, TB and chronic illnesses in children and adolescents

0830-0900	Chronic illnesses in children and adolescent health overview	
0900-1000	HIV, clinical care	
1000-1100	Tuberculosis, clinical case	
1100-1130	Tea break	
1130-1215	How to recognize multi-drug resistant tuberculosis in children (MDR-TB)	
1215-1300	Rheumatic heart disease, clinical case	
1300-1400	Lunch break	
1400-1600	Clinical practice on the Paediatric ward<br>Talking to adolescents about health concerns<br>Coaching method of teaching	All facilitators, small groups of 4-5
1600-1630	Tea break	
1630-1700	Recap, discussion, sharing of experiences	

Online appendix II

Day 5. Friday – Surgical problems and preventing infections

0800-0915	Burns in children	
0915-1030	Trauma and drowning in children	
1030-1100	Revision<br>Prevention of hospital-acquired infections<br>• The 5 moments of hand-hygiene and other infection control procedures<br>• Preventing spread of respiratory viruses, use of personal protective equipment	
1030-1100	Tea break	
1100-1200	Mortality auditing and continuous quality improvement	
1200-1300	Lunch break	
1300-1400	Quiz on Hospital Care for Children, Oxygen concentrators and Quality Improvement	
1400-1430	Test discussion and feedback	
1430	Presentation of certificates, close	

Course material available at:
https://pngpaediatricsociety.org/hospital-care-for-children-training-modules/

Duke T, *et al. Arch Dis Child* 2021; 106:224–230. doi: 10.1136/archdischild-2020-320107
