# Supplementary material for: Solar-powered oxygen, quality improvement and child pneumonia deaths: a large-scale effectiveness study
Source: Arch Dis Child. 2020 Oct 16;106(3):224–30. doi: 10.1136/archdischild-2020-320107 (PMC7907560; doi:10.1136/archdischild-2020-320107)
Supplement: Supplementary data [file archdischild-2020-320107supp003.pdf]

	Era	Paediatric admissions		Paediatric deaths		Pneumonia admissions		Pneumonia deaths		Births		Neonatal deaths		Paediatric referrals	
**Health facilities**		Number	Years	Number	Years	Number	Years	Number	Years	Number	Years	Number	Years	Number	Years
Tinsley health centre	Pre	475	3	13	3	226	3	1	3	638	3	9	3	26	3
Tinsley health centre	Post	1074	4	14	4	234	4	3	4	840	4	8	4	50	4
Togoba health centre	Pre									737	3	4	3	64	3
Togoba health centre	Post									377	4	4	4		0
Keripia health sub-centre	Post	108	4	1	4	78	4	1	4	196	4	0	4	10	4
Keripia health sub-centre	Pre	42	3	0	3	26	3	0	3	178	3	3	3	18	3
Kindeng health centre	Pre	257	3	0	3	82	3	0	3	251	3	2	3	21	3
Kindeng health centre	Post	6	3	0	3	2	2	0	2	294	4	2	4	0	3
Sigmil health sub-centre	Pre	85	3	22	3	67	3	7	3	210	3	10	3	54	3
Sigmil health sub-centre	Post	213	4	0	4	114	4	0	4	325	4	0	4	10	4
Nondugul health centre	Pre	233	3	9	3	46	3	1	3	486	3	1	3	25	3
Nondugul health centre	Post	230	4	0	4	115	4	0	4	272	4	0	4	0	4
Chuave health centre	Pre	695	3	3	3	351	3	3	3	893	3	7	3	51	3
Chuave health centre	Post	664	4	5	4	344	4	1	4	865	4	2	4	26	4
Kerowagi health centre	Pre	1075	3	2	3	570	3	1	3	649	3	4	3	35	3
Kerowagi health centre	Post	853	4	2	4	413	4	1	4	692	4	1	4	34	4
Mingende rural hospital	Pre	2711	3	84	3	611	3	27	3	2398	3	26	3	371	3
Mingende rural hospital	Post	2083	4	25	4	654	4	3	4	1553	4	9	4	155	4
Goglme health centre	Pre	299	3	5	3	157	3	3	3	421	3	4	3	32	3
Goglme health centre	Post	154	4	2	4	58	4	0	4	266	4	0	4	9	4
Gembogl health centre	Pre	216	3	8	3	100	3	5	3	343	3	4	3	15	3
Gembogl health centre	Post	220	4	5	4	124	4	2	4	411	4	0	4	11	4
Gumine health centre	Pre	879	3	8	3	404	3	5	3	542	3	12	3	64	3
Gumine health centre	Post	903	4	8	4	450	4	0	4	529	4	1	4	61	4
Laiagam health centre	Pre	39	3	8	3	31	3	1	3	579	3	13	3	13	3
Laiagam health centre	Post	18	1	0	1	12	1	0	1	1054	4	0	4	0	1
St Mary's Yapum health centre	Pre	144	2	18	2	65	2	12	2	178	2	2	2	29	2
St Mary's Yapum health centre	Post	96	4	7	4	47	3	1	3	166	4	4	4	96	4
Yampu health centre	Pre	775	2	37	2	220	2	16	2	570	2	4	2	66	2
Yampu health centre	Post	1306	4	26	4	321	4	6	4	526	3	5	3	0	4
Mambisanda district hospital	Pre	1149	3	27	3	403	3	17	3	1302	3	7	3	59	3

Mambisanda district hospital	Post	865	4	28	4	178	4	4	4	116	1	1	1	27	4
Kompiam district hospital	Pre	374	3	27	3	142	3	16	3	402	3	8	3	30	3
Kompiam district hospital	Post	471	3	5	3	110	3	0	3	312	4	7	4	0	3
Piaime health centre	Pre	1890	3	89	3									81	3
Piaime health centre	Post	51	1	1	1									2	1
Ialibu district hospital	Pre	556	3	12	3	194	3	7	3	780	3	4	3	163	3
Ialibu district hospital	Post	1025	4	34	4	350	4	8	4	1361	4	10	4	58	4
Wabag hospital	Pre	2231	3	149	3	932	3	71	3	4784	3	31	3	120	3
Wabag hospital	Post	3228	4	213	4	876	4	24	4	5008	4	64	4	25	4
Pomio health centre	Pre	75	2	16	2	45	2	5	2	85	2	9	2	35	2
Pomio health centre	Post	129	4	3	4	34	4	0	4	244	4	2	4	2	4
Det health centre	Pre	244	3	7	3	109	3	3	3	562	3	2	3	73	3
Det health centre	Post	482	4	12	4	200	4	3	4	447	4	4	4	14	4
Kaupena health centre	Pre	766	3	11	3	359	3	3	3	583	3	2	3	132	3
Kaupena health centre	Post	485	4	5	4	206	4	2	4	538	4	2	4	18	4
Kagua health centre	Pre	57	3	4	3	23	3	3	3	225	3	1	3	30	3
Kagua health centre	Post	92	4	1	4	29	4	0	4	295	4	0	4	4	4
Pangia health centre	Pre	303	3	9	3	173	3	7	3					110	3
Pangia health centre	Post	25	1	0	1	6	1	0	1					1	1
Kundiawa hospital	Pre	7279	4	502	4	1560	4	88	4	4176	3	108	3	1	3
Kundiawa hospital	Post	3866	3	238	3	792	3	22	3	3401	3	136	3		0
Kainantu district hospital	Pre	576	3	8	3	472	3	2	3	2618	3	3	3	24	3
Kainantu district hospital	Post	2652	4	119	4	977	4	37	4	4216	4	23	4	136	4
Lufa health centre	Pre	292	3	8	3	153	3	5	3	240	3	2	3	17	3
Lufa health centre	Post	292	4	4	4	127	4	1	4	299	4	2	4	17	4
Okapa health centre	Pre	286	3	4	3	180	3	2	3	260	3	2	3	28	3
Okapa health centre	Post	205	4	0	4	96	4	0	4	355	4	0	4	7	4
Onamuga health centre	Pre	688	3	18	3	297	3	15	3	485	3	7	3	31	3
Onamuga health centre	Post	512	4	12	4	291	4	4	4	573	4	2	4	38	4
Kassam health centre	Pre	211	3	5	3	107	3	3	3	613	3	2	3	17	3
Kassam health centre	Post	116	4	10	4	36	4	3	4	451	4	3	4	10	4
Obura health centre	Pre	317	3	6	3	216	3	4	3	188	3	0	3	34	3
Obura health centre	Post	148	4	6	4	76	4	1	4	247	4	4	4	15	4

BMJ Publishing Group Limited (BMJ) disclaims all liability and responsibility arising from any reliance placed on this supplemental material which has been supplied by the author(s)

Kesevaka health centre	Pre	98	2	6	2	38	2	2	2	79	3	0	3	41	3
Kesevaka health centre	Post	16	3	2	3	8	2	0	2	150	4	2	4	1	3
Kwikila rural hospital	Pre	123	3	7	3	33	3	1	3	2102	3	1	3	22	3
Kwikila rural hospital	Post	307	4	1	4	53	4	0	4	1252	4	3	4	46	4
Gaubin rural hospital	Pre	870	3	74	3	287	3	18	3	1223	3	8	3	13	3
Gaubin rural hospital	Post	1096	4	38	4	407	4	10	4	1221	4	16	4	35	4
Veifa rural hospital	Pre	473	2	6	2	319	2	0	2	663	2	0	2	25	2
Veifa rural hospital	Post	379	2	8	2	151	2	0	2	406	2	3	2	20	2
Vanimo hospital	Pre	2424	4	159	4	431	4	14	4	3190	3	68	3	1	3
Vanimo hospital	Post	1065	2	21	2	135	2	14	2	1518	2	29	2		0
Gerehu hospital	Pre	1951	2	21	2	799	2	9	2					364	2
Gerehu hospital	Post	1731	2	11	2	601	2	2	2					216	2
**Totals**		**58324**	**236**	**2259**	**236**	**18933**	**229**	**530**	**229**	**64409**	**232**	**719**	**232**	**3397**	

Online appendix III. Data on overall paediatric admissions, pneumonia admissions, births and neonatal deaths, and referrals, by hospital and by era.
